# A Novel Peptide, Nicotinyl–Isoleucine–Valine–Histidine (NA–IVH), Promotes Antioxidant Gene Expression and Wound Healing in HaCaT Cells

**DOI:** 10.3390/md16080262

**Published:** 2018-08-01

**Authors:** Dong Hwee Son, Dong Joo Yang, Ji Su Sun, Seul Ki Kim, Namju Kang, Jung Yun Kang, Yun-Hee Choi, Jeong Hun Lee, Sang Hyun Moh, Dong Min Shin, Ki Woo Kim

**Affiliations:** 1 Department of Oral Biology, BK21 PLUS, Yonsei University College of Dentistry, Seoul 03722, Korea; donghweeson@gmail.com (D.H.S.); YDJ1991@yuhs.ac (D.J.Y.); SUNJISU@yuhs.ac (J.S.S.); KIMSG1126@yuhs.ac (S.K.K.); NJKANG@yuhs.ac (N.K.); HANNAHKANG77@yuhs.ac (J.Y.K.); YHCHOI1975@yuhs.ac (Y.-H.C.); 2 Department of Global Medical Science, Yonsei University Wonju College of Medicine, Wonju 26426, Korea; 3 Anti-Aging Research Institute of BIO-FD&C Co. Ltd., Incheon 21990, Korea; jhlee@biofdnc.com (J.H.L.); shmoh@biofdnc.com (S.H.M.)

**Keywords:** nicotinamide, isoleucine-valine-histidine, antioxidant, nuclear factor (erythroid-derived 2)-like factor, wound-healing

## Abstract

Nicotinamide (NA), a water-soluble vitamin B_3_, has been shown to exert cellular-protective effects against reactive oxygen species (ROS). In order to improve the cellular-protective effects of NA, we synthesized a novel compound, nicotinyl–isoleucine–valine–histidine (NA–IVH), by combining NA with jellyfish peptides’ IVH. In the present study, we examined the cellular-protective effects of the novel synthetic nicotinyl-peptide, NA–IVH. We found that NA–IVH enhances the radical scavenging activity with a robust increase of the nuclear factor (erythroid-derived 2)-like factor (Nrf2) expression in human HaCaT keratinocytes. In addition, NA–IVH protected the cells from hydrogen peroxide (H_2_O_2_)-induced cell death. Interestingly, NA–IVH exhibited an improved wound-healing effect in a high glucose condition, possibly through the regulation of reactive oxygen species (ROS). Collectively, our results imply that a novel nicotinyl-peptide, NA–IVH, has a wound-healing effect in a hyperglycemic condition, possibly by modulating excessive ROS.

## 1. Introduction

Wound-healing is a multidimensional healing process involving various physiological regulations, by which the skin responses to wounds in order to repair itself. It is believed that once the skin is wounded, it begins a set of intrinsic responses, in which the skin undergoes various phases, including the hemostasis, inflammatory, and proliferative phases [1]. After a wound has been made, the primary response involves the inhibition of further rupture, thereby minimizing the loss of blood. During the second phase, the damaged tissues, bacteria, and pathogens are removed by the inflammatory cells [2]. However, during the inflammatory phase, large amounts of reactive oxygen species (ROS) are produced by the NADPH oxidase, an enzyme highly expressed in inflammatory cells [3,4]. Together, it has been reported that antimicrobial peptides also play a critical role in the wound healing processes [5,6,7].

It has been suggested that when present in an adequate quantity, ROS play various roles in the normal physiological wound-healing process, including bacterial killing and epithelization, and the beneficial role of ROS in the wound-healing process is further supported by a previous finding, which has suggested that wound-healing is impaired in the hypoxia condition [8]. However, excessive ROS induction has been linked with the activation of pro-apoptotic proteins, leading to cell death and necrosis [9]. For instance, an elevation of ROS levels was observed in the chronic and non-healing wounds in vivo, suggesting a possibility that excessive ROS might be detrimental to the wound-healing process [4]. Moreover, excessive levels of ROS have been linked with hyperglycemic complications, such as diabetic ulcers, and several efforts against increasing the prevalence of such disorder have been made through the regulation of excessive ROS levels [10,11]. Altogether, the homeostatic control of the cellular ROS levels may play a pivotal role in the regulation of the wound-healing process [12].

Nicotinamide (NA), a water-soluble vitamin B_3_, has been reported to enhance DNA repair and prevent ultraviolet (UV)-induced immunosuppression, thereby protecting the skin against photocarcinogenesis caused by te ROS generated by the UV [13,14,15,16,17,18,19]. Moreover, in vitro and in vivo studies have demonstrated that NA possesses antioxidant properties as well [20,21]. The NA has been incorporated into topical ointments targeted for a wide range of dermatological disorders [22,23,24]. Although there are concerns regarding the worldwide increase of the gelatinous biomass of jellyfish, an increasing number of results also showed that the jellyfish could be used as a new source of marine drugs [25,26]. In this regard, we isolated a peptide, isoleucine–valine–histidine (IVH), from a jellyfish and synthesized a novel molecule, NA–IVH, by conjugating NA to IVH, in orer to enhance the cellular-protective effects of NA. The IVH peptides that have been extracted from a jellyfish and that has exhibited several beneficial effects, including the antioxidant function [27,28,29,30,31,32].

In the present study, we analyzed the potential physiological effects of the synthetic NA–IVH in the human HaCaT keratinocytes.

## 2. Results

### 2.1. Synthesis of Nicotinyl–Isoleucine–Valine–Histidine (NA–IVH)

**Figure 1:**
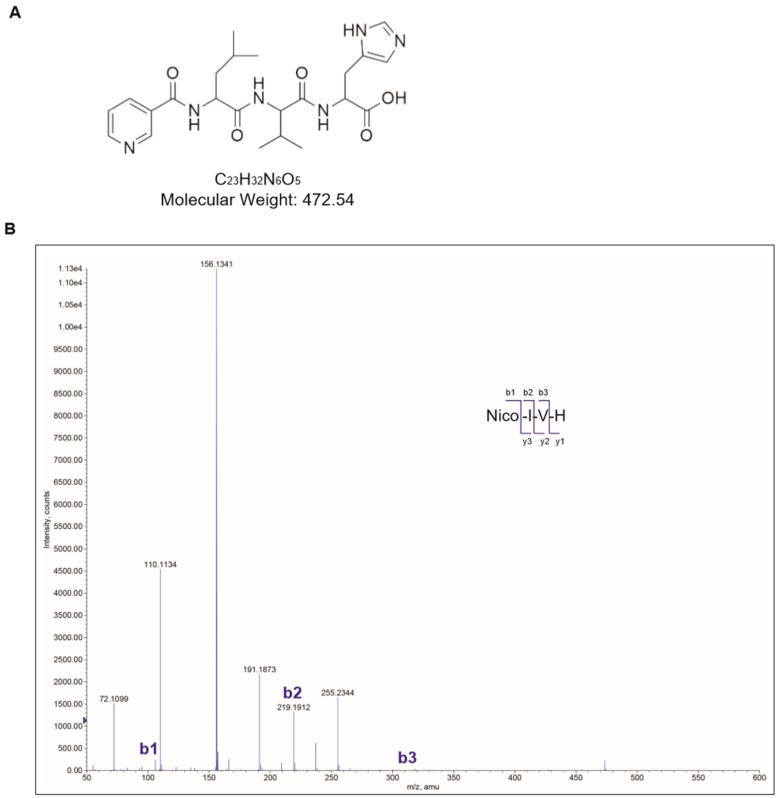
Chemical properties of nicotinyl–isoleucine–valine–histidine (NA–IVH). (**A**) Chemical structure of NA–IVH. (**B**) Mass spectrometry analysis of NA–IVH.

We analyzed the extracts of jellyfish using preparative gel electrophoresis and RP-high-performance liquid chromatography (HPLC) to determine the IVH as a bioactive and effective compound. NA–IVH was synthesized by the general hydroxybenzotriazole (HOBt)-diisopropylcarbodiimide (DIC)-mediated solid-phase peptide synthesis (SPPS) protocol [33], and was purified using high-performance liquid chromatography (HPLC). The purity of the NA–IVH was increased up to 95% after the HPLC purification. The molecular weight and chemical structure were determined by a matrix-assisted laser desorption/ionization-time of flight (MALDI-TOF) mass spectrometry assay (Figure 1A,B).

### 2.2. Cytotoxicity and Antioxidant Activity of NA–IVH

**Figure 2:**
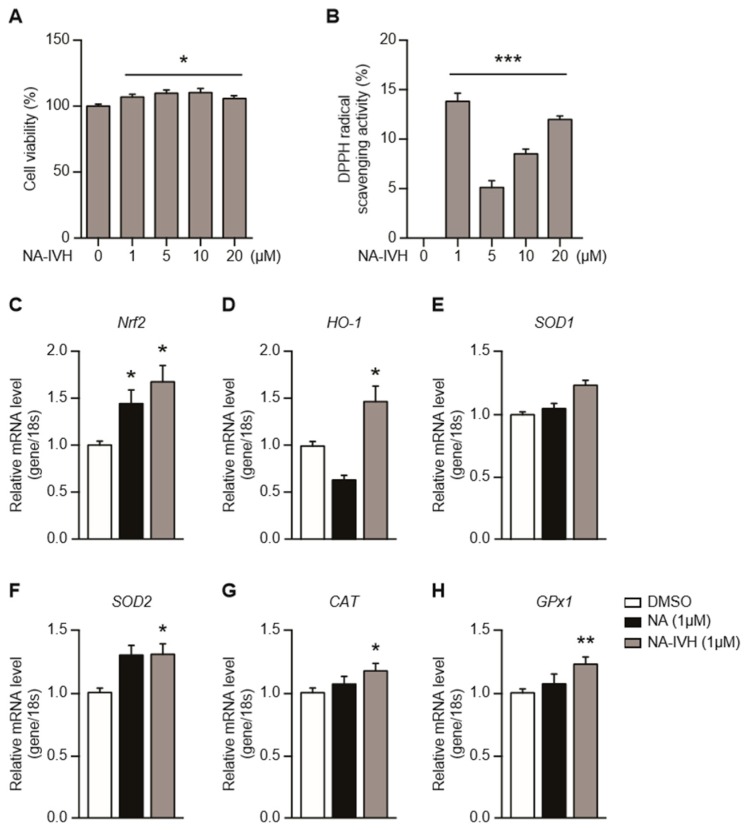
NA–IVH increases expression of antioxidant genes in HaCaT cells. (**A**) Dose-dependent cell viability of NA–IVH was assayed using MTT (3-(4,5-Dimethylthiazol-2-yl)-2,5-Diphenyltetrazolium Bromide). (**B**) Reactive oxygen species (ROS) scavenging effect of NA–IVH. (**C**–**H**) Expressions of anti-oxidant genes were measured using real-time quantitative PCR. The results are expressed as mean ± SEM (* *p* < 0.05, ** *p* < 0.01, *** *p* < 0.001, one-way analysis of variance (ANOVA), or Student’s *t*-test).

To examine the cytotoxic effect of NA–IVH, we performed a 3-(4,5-dimethylthiazol-2-yl)-2,5-diphenyltetrazoliumbromide (MTT) assay using HaCaT cells. The cells were treated with NA–IVH at different doses (0, 1, 5, 10, and 20 µM) for 24 h. After the treatment, the cell culture media was replaced with media containing 100 µL of MTT solution (0.5 mg/mL in DMSO), and was further incubated for 2 h until the MTT formazan was precipitated. As shown in Figure 2A, the NA–IVH treatment at varying concentrations did not demonstrate the cytotoxicity in HaCaT cells. To examine the antioxidant activity of NA–IVH, the DPPH (2,2-diphenyl-1-picrylhydrazyl) radical scavenging assay was used by adding 0.1 mM of a DPPH solution to each sample for 30 min before the absorbance measurement. Our data demonstrated that the NA–IVH at the concentration of 1 µM induced the most efficient antioxidant activity (Figure 2B). In response to the accumulating ROS, cells require an efficient antioxidant activity achieved by the activity of ROS-detoxifying enzymes and proteins. To determine the antioxidant function, the expressions of the antioxidant genes were examined upon the NA–IVH treatment. As shown in Figure 2C, the NA–IVH treatment for 24 h increased the Nrf2 expression. Next, we examined the downstream genes of Nrf2, including heme oxygenase-1 (HO-1), superoxide dismutase 1 and 2 (SOD 1 and 2), Catalase (CAT), and glutathione peroxidase 1 (GP×1) (Figure 2D–H). Interestingly, all of the genes examined showed a significant increase, and the increase was more distinctive than that of the original form of NA (Figure 2C–H). These data indicate that NA–IVH has the potential to regulate oxidative stress through the activation of antioxidant genes.

### 2.3. Protective Role of NA–IVH on ROS-Induced Cytotoxicity

**Figure 3:**
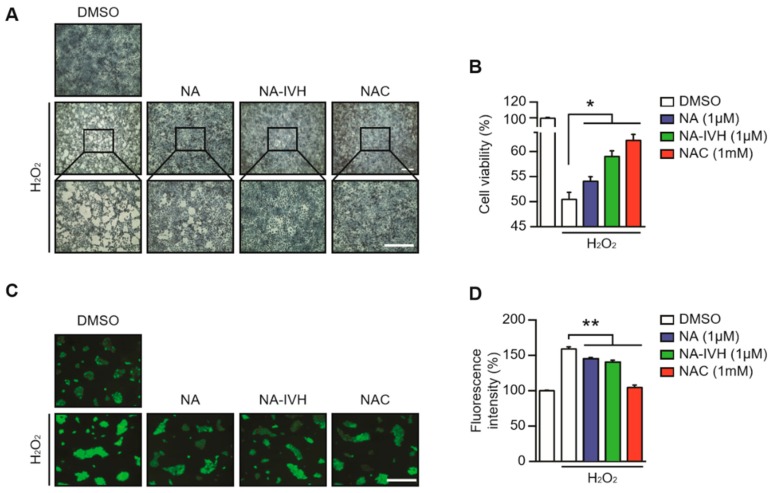
Antioxidant role of NA–IVH in HaCaT cells. (**A**) Antioxidant effect of NA–IVH in the presence of H_2_O_2_ was monitored using methylene blue staining. (**B**) Effect of NA–IVH on cell viability in the presence of H_2_O_2_. (**C**) ROS scavenging effect of NA–IVH in the presence of H_2_O_2_. (**D**) Fluorescence intensity (%) from (**C**). The results are expressed as mean ± SEM (* *p* < 0.05, ** *p* < 0.01 against vehicle with H_2_O_2_ treatment, one-way ANOVA). Scale bar 0.5 mm.

Next, we examined the antioxidant role of NA–IVH upon the treatment of 1 mM of H_2_O_2_. HaCaT cells were pretreated with either dimethyl sulfoxide (DMSO), NA, NA–IVH, or n-acetyl cysteine (NAC) for 2 h before H_2_O_2_ introduction. Methylene blue staining and MTT assay were performed after H_2_O_2_ treatment for 21 h. The cell death induced by H_2_O_2_ was significantly reduced by the pretreatment of NA–IVH (Figure 3A,B). Furthermore, the pretreatment of NA–IVH markedly reduced the H_2_O_2_-induced ROS generation in the HaCaT cells as measured by 2’,7’ –dichlorofluorescin diacetate (DCFDA), suggesting NA–IVH as a potential ROS-scavenging molecule (Figure 3C,D). Intriguingly, NA–IVH also showed a more robust antioxidant activity compared to that of NA, implying that the conjugation between NA and IVH might enhance its original antioxidant function (Figure 3).

### 2.4. Improved Wound-Healing Effect of NA–IVH in High Glucose Condition

**Figure 4:**
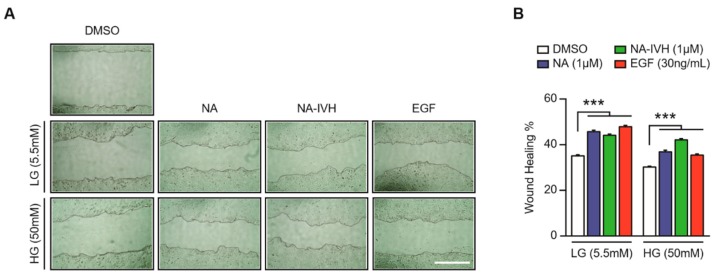
NA–IVH accelerates wound-healing in a high glucose (HG) condition. (**A**) Wound-healing effect of either DMSO, NA, NA–IVH or epidermal growth factor (EGF) in low (5.5 mM) or high (50 mM) glucose condition. LG—low glucose; HG—high glucose. (**B**) Wound-healing (%) was measured in either a low (5.5 mM) or high (50 mM) glucose condition. The results are expressed as mean ± SEM (*** *p* < 0.001, one-way ANOVA). Scale bar 0.5 mm.

A recent study demonstrated that the Nrf2 expression might be associated with the wound-healing process in a diabetic mouse model, suggesting a potential contribution of NA–IVH in the wound-healing process in a high glucose (HG) condition, given that NA–IVH increased the expression of Nrf2 [34]. Hence, we performed a wound-healing assay, in which the HaCaT cells cultured in HG condition were wounded prior to a treatment of either DMSO, NA, NA–IVH, or epidermal growth factor (EGF) for 24 h. Although the cells in the HG media showed a delayed wound closure compared to that of the cells in the low-glucose (LG) media, the cells treated with NA–IVH in the HG media showed an accelerated wound closure when compared to that of the vehicle-treated (Figure 4A,B). Moreover, it is noteworthy that NA–IVH in the HG condition showed a greater wound-healing efficacy, demonstrated by a narrower wound gap than that of the NA treated cells. This result indicates that NA–IVH is capable of accelerating the wound-healing of the HaCaT cells in the diabetic condition.

## 3. Discussion

NA has been well-recognized as a cellular protective molecule for its antioxidant properties [20,21], and efforts to produce a novel NA peptide that retains the beneficial effects of NA with a greater potency in carrying out its effects have been made [35]. In the current study, we synthesized a novel nicotinyl-derivative, NA–IVH, which retains the antioxidant and wound-healing properties of NA with a greater efficacy. It has been reported that the Nrf2 expression is modulated by the ROS in keratinocytes [36,37,38,39,40]. The Nrf2 is widely known for its orchestration of the transcription of endogenous antioxidant genes via an antioxidant response element (ARE) [41]. In this study, we found that Nrf2 was increased upon NA–IVH treatment and led to the activation of its downstream antioxidant genes, including HO-1, SOD1 and 2, CAT, and GPx1 in HaCaT cells (Figure 2), suggesting that the antioxidant and anti-cytotoxic effect of NA–IVH could at least partially come from the direct regulation of antioxidant genes. In addition, Nrf2 has been indicated as a target gene of keratinocyte growth factor (KGF), and is upregulated in the wounded epidermis [42,43,44,45], indicating a possible involvement of Nrf2 in the wound-healing process on the skin. However, recent evidence demonstrated that Nrf2-mediated gene expression in keratinocytes is unnecessary for wound-healing, at least under normal conditions [43]. Hence, whether Nrf2 is required for wound-healing under varying circumstances requires further studies.

Interestingly, Long et al. demonstrated that the pharmacological activation of Nrf2 pathway significantly improved diabetic wound-healing, suggesting a possible involvement of the Nrf2 activators as treatments for diabetic skin ulcers [34]. Moreover, mice lacking Nrf2 demonstrated a slowed wound-healing process, due to the deprivation of efficient antioxidant activity [34]. Environmental challenges, such as UV irradiation, toxic chemicals, and mechanical wounding, result in increased reactive oxygen species (ROS), inflammation, skin aging, and cancer development [46]. Although ROS are generated during the normal metabolic processes and are required for regular cellular signaling, excessive ROS levels are suggested to cause severe cellular damage, leading to disease development [46,47]. Hence, the development of an efficient ROS defensive mechanism on the skin has been of great interest. Although other studies suggest that presence of ROS is supportive of wound-healing [5,35], the excessive production of ROS leads to oxidative stress, causing severe cell damage and cell death [4]. Hence, the homeostatic regulation of ROS might be essential for an efficient wound-healing process. Therefore, a novel NA–IVH having an antioxidant and wound-healing function could work as homeostatic component in the wound-healing process.

Wound closure upon NA and NA–IVH treatment in a low glucose (LG) condition did not demonstrate a significant difference between the two. However, we found that the accelerated wound-healing process when NA and NA–IVH were introduced to wounded cells grown in a high glucose (HG) condition (Figure 4). Therefore, our study demonstrates that a novel nicotinyl-peptide, NA–IVH, possesses a wound-healing effect in the hyperglycemic condition, possibly by modulating excessive ROS.

## 4. Materials and Methods

### 4.1. Synthesis and Purification of Nicotinic Acid–IVH

Amino acids and nicotinic acid were coupled to the resin by general 1-hydroxybenzotriazole-diisopropylcarbodiimide (HOBt-DIC) mediated solid-phase peptide synthesis (SPPS) protocol [33] and then purified by reverse-phase HPLC (Waters, MA, USA; pump 600E, UV-484 detector, Gemini RP-C18 column 250 × 21.2 mm) in a gradient of acetonitrile in 0.1% trifluoroacetic acid. A MALDI-TOF mass spectrometry assay was performed to determine the quality of the synthetic NA–IVH. All of the mass spectrometry (MS) or MS/MS experiments were performed on a mass spectrometer (Q-Star XL, AB Sciex, Foster City, CA, USA) with a turboionspray source. The electrospray ionization (ESI) ion source parameters were a spray voltage 5.0 kV, curtain gas 25 L/min, and nebulizer gas 20 L/min. Synthetic peptides were confirmed for the molecular weights in full scan mode, and then sequenced by MS/MS scans. The collision energies were varied from 20 to 40 to efficiently get the fragments. The results were manually interpreted by Analyst QS 1.1 with Bioanalyst extensions (or all MS or MS/MS experiments followed the methods described in previous report [35]).

### 4.2. Cell Culture and Reagents

Human keratinocytes, HaCaT cells, obtained from Dr. Fusenig, Deutsches Krebsforschungszentrum, Heidelberg, Germany [48], were grown in Dulbecco’s modified Eagle’s medium (DMEM, low glucose, Hyclone, Logan, UT, USA) containing a 10% heat inactivated fetal bovine serum (FBS) at 37 °C in 5% CO_2_. The high glucose (HG) condition DMEM was made with an addition of 1 M glucose or 0.5 M mannitol to Dulbecco’s modified Eagle’s medium (DMEM, low glucose, Hyclone, Logan, UT, USA) in order to produce high glucose in the cell culture system or to adjust osmosis, respectively [49]. Epidermal growth factor (EGF), n-acetyl cysteine (NAC), H_2_O_2_, glucose, and mannitol were purchased from Sigma-Aldrich (St. Louis, MO, USA).

### 4.3. Cell Viability Assay

An MTT assay was performed using the 3-(4,5-dimethylthiazol-2-yl)-2,5-diphenyltetrazoliumbromide (MTT) based cell viability and proliferation Kit 1 (Sigma-Aldrich, St. Louis, MO, USA), following the suggested protocol. In brief, 3 × 10^4^ cells were seeded in 96-well microplates and cultured for 24 h prior to treatment. The cells were treated with DMSO, different concentrations of NA–IVH (0, 1, 5, 10, and 20 µM), or N-Acetyl cysteine (NAC, 1 mM), with/without 1 mM H_2_O_2_, and incubated for 24 h, then, the media was replaced with 100 µL of MTT solution (0.5 mg/mL in DMEM), in which the cells were incubated at 37 °C for 4 h. The MTT solution was removed and MTT formazan dissolved in 100 µL of dimethyl sulfoxide (DMSO) was added. The absorbance was measured at 540 nm using a microtiter plate reader. For the cell viability visualization, HaCaT cells were pre-treated with or without either NA (1 µM), NA–IVH (1 µM), or NAC (1 mM) for 2 h, and were the incubated with or without 1 mM of H_2_O_2_ for 21 h. The media was discarded, then the cells were washed with PBS and fixed using 95% (*v*/*v*) ethanol for 5 min. After fixation, the cells were washed with PBS and stained using 2 mL 2% methylene blue solution for 1 min. An image was acquired at indicated time using AxioObserver FL microscope at 10 and 20× magnification.

### 4.4. DPPH Radical Scavenging Assay

A DPPH (2,2-diphenyl-1-picrylhydrazyl) radical scavenging assay was used to determine the radical scavenging effects of the antioxidant compounds [50,51]. The DPPH (Sigma-Aldrich, MO, USA) solutions were prepared in ethanol as a stock solution, and diluted to 0.1 mM final working concentration. Then, 0.1 mM DPPH solution was added to 0.5 mL of each sample, and the samples were incubated at 4 °C for 30 min. After the reaction was complete, the absorbance was measured at 517 nm using a Multiskan Go Spectrophotometer (Thermo Fisher Scientific, MA, USA).

### 4.5. ROS Scavenging Assay

Intracellular ROS scavenging assays were performed by measuring the fluorescence intensity of the 5-(and-6)-chloromethyl-2′,7′-dichlorodihydrofluorescein diacetate, acetyl ester probe, which intensity is in accordance to the amount of ROS in presence. The HaCaT cells were pre-treated with or without either DMSO, NA (1 µM), NA–IVH (1 µM), or NAC (1 mM) for 24 h, and were then incubated with or without 1 mM of H_2_O_2_ for 2 h. The media was replaced with PBS containing 5 µM of 5-(and-6)-chloromethyl-2′,7′-dichlorodihydrofluorescein diacetate, acetyl ester (CM-H2DCFDA; Thermo Fisher Scientific, MA, USA) for 20 min, prior to detection. The images were captured using a IX81 inverted microscope (Olympus, Tokyo, Japan) at 2 × magnification. The images acquired were subjected to intensity measurement using Metamorph 6.1 software (Molecular Devices, Sunnyvale, CA, USA).

### 4.6. In Vitro Wound-Healing Assay

The cells were grown to confluence in 6-well plates and a scratch wound was created manually using a P100 pipet tip [52]. The HaCaT cells were treated with or without either DMSO, NA (1 μM), NA–IVH (1 μM), or EGF (30 ng/mL), for 24 h. The media was discarded and the cells were washed with PBS. The image was acquired at the indicated time using the AxioObserver FL microscope at 10× magnification. The width of the wound was measured in more than three locations and the percentage of the healing area was indicated using Image J (ver.1.51J8, Wayne Rasband National Institutes of Health, USA) software. The data was obtained from more than three independent experiments.

### 4.7. Quantitative Real-Time PCR

The total RNA was isolated from cells using a TRIzol reagent (Invitrogen, Carlsbad, CA, USA). The cDNAs were synthesized using a High Capacity cDNA Reverse Transcription Kit (Applied Biosystems, Foster City, CA, USA), in accordance with the manufacturer’s instructions, then were subjected to quantitative real-time PCR and transcription expression of the following genes that were analyzed; CAT (F; 5′-CTGAGTCTCTGCATCAGGTTT-3′, R; 5′-TCATGTGGCGATGTCCAT-3′), GP×1 (F; 5′-GCACCCTCTCTTCGCCTTC-3′, R; 5′-TCAGGCTCGATGTCAATGGTC-3′), Nrf2, (F; 5′-CAGGCTCAGTCACCTGAAACTTCT-3′, R; 5′-TCTCTGGTGTGTTCTCACATTGGG-3′), HO-1 (F; 5′-ACCCATGACACCAAGGACCAGA-3′, R; 5′-GTGTAAGGACCCATCGGAGAAGC-3′), SOD1 (F; 5′-ACGGTGGGCCAAAGGAT-3′, R; 5′-TCTTTGTCAGCAGTCACAATTGC-3′), SOD2 (F; 5′-AATCAGGATCCACTGCAAGGA-3′, R; 5′-AGGCGTGCTCCCACACATC-3′), and SPTLC2 (F; 5′-GGAGATGCTGAAGCGGAAC-3′, R; 5′-GCTGCAATAGGTCCCCAACT-3′).

## 5. Statistical Analysis

The Prism 5.0 software was utilized for all of the statistical analyses. One-way analysis of variance (ANOVA) or Student’s *t*-test was used to assess the difference between groups, and *p* < 0.05 was regarded as a statistical significance difference.
